# Combination of Endogenous Estradiol and Adipokine Leptin in Breast Cancer Risk and Prognosis Assessment in Postmenopausal Chinese Women

**DOI:** 10.3389/fendo.2021.766463

**Published:** 2021-12-14

**Authors:** Yang Luo, Han-Bing Li, Yue Zhang, Yu-Xin Wu, Di Shen, Yi-Qun Che

**Affiliations:** ^1^Department of Medical Oncology, National Cancer Center/National Clinical Research Center for Cancer/Cancer Hospital, Chinese Academy of Medical Sciences and Peking Union Medical College, Beijing, China; ^2^Department of Clinical Laboratory, National Cancer Center/National Clinical Research Center for Cancer/Cancer Hospital, Chinese Academy of Medical Sciences and Peking Union Medical College, Beijing, China; ^3^Center for Clinical Laboratory, Beijing Friendship Hospital, Capital Medical University, Beijing, China

**Keywords:** estradiol, leptin, postmenopausal, elderly patients, breast cancer

## Abstract

**Objective:**

Our study aims to clarify the role of estradiol and leptin in breast cancer risk and prognostic assessment in postmenopausal Chinese women.

**Design:**

The serum circulating estradiol and leptin level was detected by ELISA. Then the correlation between estradiol, leptin level, and clinical characteristics was analyzed using Fisher’s exact test. Next, the Kaplan-Meier model was used to analyze the association between estradiol, leptin, and prognosis of postmenopausal breast cancer patients in our cohort and the TCGA dataset.

**Setting:**

The study was conducted at the National Cancer Center, Cancer Hospital, Chinese Academy of Medical Sciences, and Peking Union Medical College.

**Patients:**

A total of 182 postmenopausal breast cancer patients and 111 healthy subjects from January 2010 to August 2010 were included in the analysis. Another 702 cases with breast cancer were retrieved from The Cancer Genome Atlas (TCGA) database for subsequent analysis.

**Main Outcome Measure:**

Serum circulating estradiol and leptin level.

**Results:**

The level of estradiol was significantly higher (*P*<0.001) but the level of leptin had no significant difference (*P* = 0.764) in postmenopausal breast cancer patients compared with healthy subjects. The level of estradiol and leptin was not significantly different between estrogen receptor (ER) positive and ER-negative groups (*P*>0.05). Estradiol was significantly correlated with tumor T stage (*P* = 0.002) and leptin was significantly associated with perineural invasion (*P* = 0.014). In addition, the disease-free survival of patients with a high level of estradiol was significantly shorter (*P* = 0.025) but leptin tended to be a protective factor for overall survival in TCGA analysis (*P* = 0.038).

**Conclusion:**

Circulating estradiol and leptin played important roles in the risk of postmenopausal breast cancer even in low-estrogen nations with an independent expression of ER status. High circulating estradiol was a poor prognostic factor and leptin may be a protection signal in Chinese postmenopausal patients with breast cancer.

## Introduction

Breast cancer is the most common tumor in women leading to a high tumor burden worldwide. In 2020, the estimated new cases of breast cancer in women are 276,480 and the estimated deaths are 42,170 in the United States, which ranks at 1st and 2nd respectively (1). The incidence rate of breast cancer in China has risen more than twice as fast as the global rates, particularly in urban areas. Meanwhile, it is also noticeable that the morbidity shows as a double peak, which means the incidence of elderly breast cancer patients is also increasing (2). However, in China, the incidence rates of breast cancer have increased over the past thirty years. Although this trend is largely attributed to changes in reproductive patterns and the use of mammography screening, the increase also reflects lifestyle changes, physical inactivity, the prevalence of obesity, and the use of menopausal hormones, etc (3).

The main circulating estrogens in the human body are estrone (E1), estradiol (E2), and estriol (E3), among which E2 is the most biologically active estrogen. In postmenopausal women, the breast cancer risk is significantly elevated with higher levels of total and free E2 (4). E2 plays a vital role in the development and progression of breast cancers through their oxidative metabolites, and by affecting cell proliferation and apoptosis through their interaction with the estrogen receptor (ER) in breast tissue (5). So far, prospective epidemiological studies have documented the association between circulating E2 and the risk of postmenopausal breast cancer and evaluated circulating hormones as potential prognostic markers for breast cancer (6, 7). However, these studies included only women from developed western countries that have a relatively high breast cancer incidence. More importantly, it has been recognized that the concentrations of E2 are comparatively low in postmenopausal Asian women, particularly in China (8). Whether E2 still has evident risk associations with breast cancer in postmenopausal Chinese populations with low estradiol levels is not known.

In postmenopausal women, adipose tissue becomes the main producer of E2 through the enzymatic conversion of androgen precursors to E2 carried out by aromatase (9). In obese women, the elevated aromatase levels led to an increase of E2, resulting in poorer treatment effects and inferior prognosis in breast cancer (10). The linkage between obesity and breast cancer was potentially built by multiple factors, including hyperglycemia, dyslipidemia, adipokines and cytokines, and the gut microbiome (11). Aberrant adipokine- and cytokine-mediated molecular signaling are the key features in obesity-associated breast cancer and leptin is one of the most important adipokines (12). It is worth mentioning that the circulating levels of leptin increase proportionally to total adipose tissue mass and leptin has been identified as a key molecule in breast cancer risk and tumor biology (13). Leptin binds to the leptin receptors on the plasma membrane to drive multiple downstream signaling cascades, such as JAK-STAT, PI3K-Akt-FoxO1, AMPK, and mTOR-S6K that involves in the cell proliferation, transformation, migration, and invasion of cancer (14, 15). However, there are substantial racial or ethnic differences in obesity rates. Compared with white women in western countries, Chinese women have a markedly lower rate of obesity (16). Therefore, the role of leptin in the Chinese population needs to be further clarified.

In this study, we initially identified the expression levels of estradiol (E2) and leptin in breast cancer patients and healthy subjects with menopause. And the relationship between E2, leptin, and clinicopathological parameters of postmenopausal patients with breast cancer was analyzed. Then, the role of E2 and leptin in the prognosis assessment of postmenopausal breast cancer patients was performed.

## Materials and Methods

### Patients

A total of 293 cases were enrolled in this study, including 182 newly diagnosed postmenopausal breast cancer patients and 111 age-matched healthy subjects. All breast cancer patients and healthy people were from the National Cancer Center/Cancer Hospital, Chinese Academy of Medical Sciences (Beijing, China). All samples were collected prior to treatment in the Cancer Hospital of the Chinese Academy of Medical Sciences between January 2010 and August 2010. All of the subjects were selected based on medical and pathology reports and followed up for 120 months. Blood samples of healthy subjects were obtained from donors in absence of any disease. The inclusion criteria of patients were as follows: ① breast cancer diagnosed by pathology; ② female; ③ aged over 55 years old and more than 1 year after menopause; ④ follicle stimulating hormone (FSH) >40U/L ⑤ no endocrine therapy or chemotherapy was performed before inclusion; ⑥ complete clinical data and follow-up records. The exclusion criteria include ① with distant metastasis of the tumor ② presence of another malignant tumor; ③ existing heart, lung, liver, kidney disease, or another severe disease. The cutoff values of body mass index (BMI) were based on follows: ① normal weight: BMI<24; ② overweight: 24≤BMI<28; ③ obesity: BMI≥28. The study was approved by the Ethics Committee of the Cancer Hospital of the Chinese Academy of Medical Sciences. All patients were provided with written informed consent prior to enrollment in this study.

### Enzyme−Linked Immunosorbent Assay

All blood samples were collected before surgery, 4mL of each venous blood samples were centrifuged at 3000g for 10 minutes, and the sera were transferred into new tubes for further experiments. Serum concentrations of E2 and leptin in 182 breast cancer patients and 111 healthy subjects were measured in duplicate by ELISA using the commercial kits (mlbio, Cat. ^#^ml038613-J and RayBiotech, Cat. ^#^1011190158). The whole processes were performed according to the manufacturer’s instructions. Subsequently, the standard solution provided by the kit was diluted to establish the calibration curve. Then, the absorbance of each sample was measured at 450 nm against the blank with an ELISA plate reader. Finally, the results were drawn on the basis of the calibration curve to quantify the target hormone and protein.

### Statistical Analysis

Statistical analyses were performed by using SPSS version 22.0 (IBM, Armonk, NY, USA) and all figures were drawn by GraphPad Prism 7.0 (Graph Pad Software, San Diego, CA, USA). The E2 and leptin levels between breast cancer patients and healthy subjects were compared by Mann Whitney test. Then, the Fisher exact test was used to compare the relationship between E2, leptin level and clinicopathological factors. Next, the Kaplan Meier survival curve was plotted according to the prognostic data to analyze the role of E2 and leptin in overall survival (OS) and disease-free survival (DFS) of breast cancer patients. All statistical tests were two-sided and a p value < 0.05 was considered statistically significant.

### TCGA Data Analysis

In order to further validate the explicit role of leptin in prognostic assessment, a total of 702 postmenopausal cases with breast cancer were retrieved from The Cancer Genome Atlas (TCGA) database for analysis. Similarly, the complete prognosis information of breast cancer patients was recorded. The Kaplan Meier survival model was applied to evaluate the effect of leptin in OS and progression-free survival (PFS) of breast cancer patients. The p value < 0.05 was considered statistically significant.

## Results

### The Basic Information of Postmenopausal Patients With Breast Cancer

A total of 182 stage I-III breast cancer patients were followed for a median of 7.7 years (interquartile range 6.0 – 8.8). The basic clinical information is shown in **Table 1**. The median age of all patients was 63 years. A total of 32 patients suffered recurrence and 6 deaths from breast cancer occurred over the follow-up period.

**Table 1:** Basic clinical characteristic of postmenopausal patients enrolled in analysis.

Features	Group	Patients(n)
Age (y)	>60	101
	55 - 60	81
Family history	Yes	15
	No	167
Pathological type	Invasive ductal carcinoma	156
	Other types	26
Stage	I-II	101
	III	81
Ki-67 index	≤20%	70
	>20%	112

### Comparison of E2 and Leptin Expression Between Postmenopausal Breast Cancer Patients and Normal Postmenopausal Women

**Figure 1:**
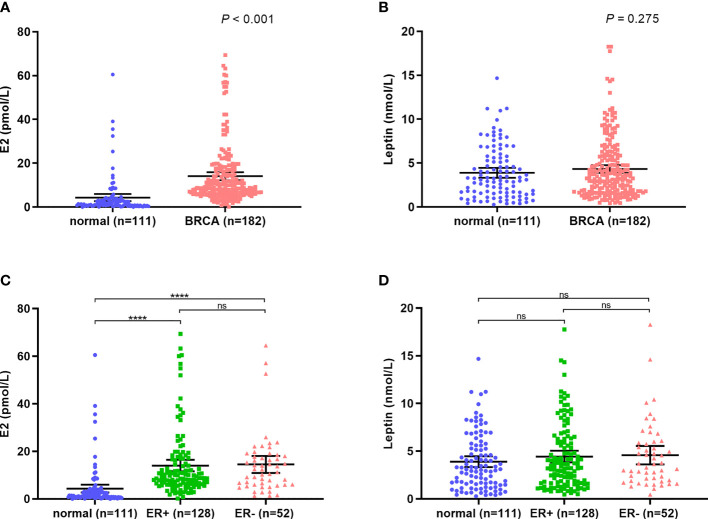
E2 and leptin levels in breast cancer patients and normal subjects. **(A)** High level of E2 in breast cancer patients compared with normal subjects; **(B)** The level of leptin showed no significant difference between breast cancer patients and normal subjects; **(C, D)** The levels of E2 and leptin are independent of ER expression. ****P < 0.0001; ns, *P* > 0.05.

The sera of a total of 293 cases, including 182 breast cancer patients and 111 healthy subjects, were detected by ELISA. E2 levels were considerably higher among postmenopausal breast cancer patients compared to healthy subjects (*P*<0.001) (**Figure 1A**). In contrast, leptin level did not differ between the two groups (*P* = 0.764) (**Figure 1B**). Interestingly, for both E2 and leptin, the levels were not significantly different between ER positive and ER negative groups (*P*>0.05) (**Figures 1C, D**).

### Relationship Between E2, Leptin, and Clinicopathological Factors

The levels of E2 and leptin in relation to patients and tumor variables were shown in **Table 2**. The results revealed that E2 levels were positively associated with T-stage (*P* = 0.002). However, there were no clear correlations between E2 levels and lymph node metastasis, perineural invasion, or intravascular cancer emboli. In terms of leptin, it was negatively associated with perineural invasion (*P*=0.014). BMI was remarkably associated with both E2 and leptin concentrations, with a positive association observed for leptin (*P* = 0.008) and a suggestive association with E2 (*P* = 0.088).

**Table 2:** Relationship between E2 and clinicopathological factors of elderly breast cancer patients.

Variables	Low E2	High E2	p value	Low leptin	High leptin	p value
Age			0.299			0.656
55-60	40	48		46	42	
>60	51	43		45	49	
BMI			0.088			**0.008**
< 24	24	37		39	22	
24-28	47	34		39	42	
> 28	20	20		13	27	
T stage			**0.002**			0.762
I	57	35		45	47	
II	33	49		41	41	
III	1	7		5	3	
Lymph node metastasis			1.000			0.549
Yes	39	38		41	36	
No	52	53		50	55	
Perineural invasion			0.332			**0.014**
Yes	7	12		15	4	
No	84	79		76	87	
Intravascular cancer emboli			0.853			0.352
Yes	19	17		21	15	
No	72	74		70	76	
ER status			0.328			0.744
positive	68	61		63	66	
negative	23	30		28	25	
PR status			0.444			0.283
positive	60	54		53	61	
negative	31	37		38	30	
HER2 status			0.549			0.764
positive	36	41		40	37	
negative	55	50		51	54	

BMI, body mass index; ER, estrogen receptor; PR, progesterone receptor; HER2, human epidermal growth factor receptor-2.

Bold, p＜0.05.

### High Level of E2 Was Associated With Inferior DFS for Breast Cancer Patients

**Figure 2:**
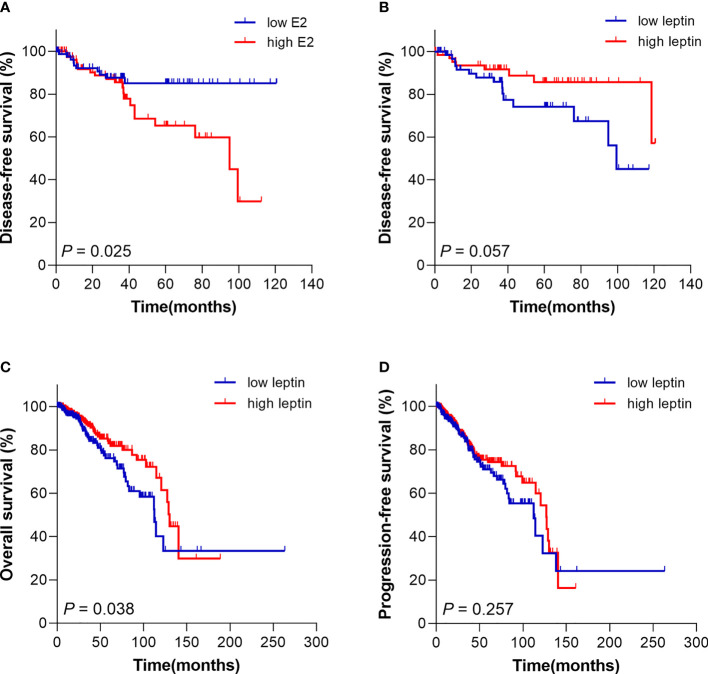
Kaplan Meier survival curve for prognosis analysis of breast cancer patients with different E2 and leptin levels. **(A)** The disease-free survival of patients with high E2 level was poorer compared with patients with low E2 level. **(B)** High leptin level trended to be a protective signal for disease-free survival of patients. **(C)** Patients from TCGA database with high leptin level had superior overall survival than patients with low leptin level. **(D)** The progression-free survival of patients from TCGA database with different leptin level presented no significant difference.

The Kaplan Meier survival model was performed to evaluate the effect of E2 and leptin on prognosis of breast cancer patients. The results demonstrated that E2 and leptin had no significant correlation with OS in breast cancer patients (*P* = 0.620 for E2, *P* = 0.954 for leptin). However, the DFS of the patients with high level of E2 was significantly shorter than those with low level of E2 (*P* = 0.025) (**Figure 2A**). On the other hand, leptin has a trend to be a protective factor in DFS, which revealed that patients with high level of leptin tended to have better DFS time (*P* = 0.057) (**Figure 2B**).

### High Level of Leptin Was Associated With Superior Survival in Breast Cancer Patients From TCGA Database

Further, in order to clarify the value of leptin in prognosis assessment and verify the results of our analysis, a total of 702 postmenopausal cases with breast cancer were retrieved from The Cancer Genome Atlas (TCGA) database for prognostic analysis. The results showed that the OS of patients with high leptin level was superior compared with those with low level of leptin (*P* = 0.038) (**Figure 2C**). The results from TCGA were consistent with our analysis above. Nevertheless, the PFS presented no obvious difference in different groups (*P* = 0.257) (**Figure 2D**).

## Discussion

Previous research has shown that the circulating E1 and E2 developed the incidence of breast cancer (17). Our study found that the E2 levels of postmenopausal Chinese patients with breast cancer were significantly higher than that of healthy subjects, although the concentrations of E2 are comparatively low in the Chinese population. In addition, given the effects of E2 on the breast, previous views might follow that the associations between circulating E2 and breast carcinogenesis would be stronger for ER-positive cancers. Nevertheless, we found no significant heterogeneity of the level of E2 by tumor ER status.

With evidence indicating that E2 is an important reason for the etiology of breast cancer, a lot of existed breast cancer risk factors that have been proposed to influence hazard *via* effects on E2 have drawn a great deal of attention in the last two decades. Obesity, defined as BMI> 30 kg/m^2^, elevated the risk of postmenopausal breast cancer through the higher circulating levels of E2 synthesized in the adipose tissue of obese women (7). However, the findings presented inconsistent and in previous population studies of postmenopausal women, BMI explains only a small to moderate proportion of variance in the E2 concentration of postmenopausal women (18). More importantly, considerable racial differences existed with obesity rates ranging from 2.6% for Chinese to 34.9% for American (19). Thus, other substitutive factors must be explored in population E2 differences.

Current study has demonstrated that E2 induced leptin expression by overexpression of p65 subunit, the active form of NF-κB (20), and leptin upregulated COX-2 to promote aromatase expression, so as to increase the production of E2 (21). Meanwhile, the level of leptin elevated with the increase of adipose tissue (22), and it was a key factor in the regulation of body weight and energy homeostasis (23). Therefore, we identified leptin, which was mainly produced by adipose tissue and recognized as a critical adipokine linking obesity to cancer, as a candidate molecule to analyze its role in breast cancer. However, the results of our study indicated that the level of leptin had no significant difference between the breast cancer patients and normal subjects, but leptin was significantly related to body weight and BMI.

While the positive correlation between circulating E2 and breast cancer risk has been well confirmed (24), there is likewise little evidence for how circulating E2 may relate to tumor characteristics in elderly patients. Our results showed that the level of E2 was significantly correlated with tumor size in postmenopausal women with breast cancer. The prior study has revealed that increased expression of leptin is associated with oncogenesis and progression of breast cancer (25). The reasons for leptin advanced the development of breast cancer may be that leptin promoted the growth and proliferation and enhanced the invasion and metastasis of breast cancer cells via activating the JAK/STAT3 and PI3K/AKT signaling pathways (26, 27). On the contrary, our finding showed that leptin was negatively correlated with neural invasion in breast cancer patients. Further studies were needed to clarify the deep mechanism of these molecules in regulating breast cancer.

Then, in order to eliminate how E2 and leptin affected the prognosis of breast cancer patients, the relationship between E2, leptin, OS, and disease-free survival was discussed. Disease-free survival (DFS) means the time from the beginning of randomized study to disease recurrence or death for any reason. DFS is most commonly used in the study of adjuvant therapy after radical surgery or radiotherapy in cancer patients. The DFS rate is a helpful indication of how effective a particular treatment is in clinical practice. For our cohort, all of the breast cancer patients enrolled experienced surgical treatment and advanced stage patients with distant metastasis were excluded, so DFS rather than PFS is more suitable for prognostic evaluation in our study. Our findings suggested that higher postmenopausal E2 levels in serum were associated with moderately worse DFS but not with OS in breast cancer. It was in accordance to prior studies (28), however others have not observed an association (29). Meanwhile, our study revealed that leptin presented a trend of protection in the prognostic evaluation for patients with high leptin level may have a superior DFS. Conversely, a study presented that leptin was significantly related to poor prognosis in overall and tamoxifen-treated breast cancer patients, and may contribute to tamoxifen resistance through inducing increased nuclear expression of ERα (30).

With these inconsistencies, we downloaded data from the TCGA database to determine the correlation between leptin and prognosis of breast cancer patients. It was shown that patients with high levels of leptin had superior OS compared with those with low levels of leptin. This phenomenon may be due to the “obesity paradox”, which means the risk of mortality of cancer is significantly reduced for BMI values above normal weight, yet this effect is lost when BMI increases to morbid obesity levels (31). This phenomenon can be explained in three aspects: obesity may affect the biological behavior of tumors; obese patients may respond better to therapy; the excess of adipose tissue may be an energy store which ensures further surviving time in some patients (32). In the Chinese population, relatively few people are extremely obese, and moderately increased BMI trended to be a protective factor in prognosis assessment of breast cancer.

Our study exhibited that E2 developed the risk of breast cancer, certain limitations to our study should be taken into consideration, chief among which is the sample size of the present study was relatively small due to the limited number of elderly breast cancer patients. An additional potential limitation is that most TGCA cases were white women, and their characteristics may not individually match to each case in our cohort. Therefore, the bias of ethnic groups may exist. In addition, considering the complicated risk factors that affect breast cancer in Chinese women, reproductive and hormonal factors, obesity and low levels of physical activity, and dietary patterns should have been comprehensively analyzed, and estrogen, estrogen receptor, androgen, androgen receptor, and leptin receptor values should be measured to get more complete information. In the future, large scale multicenter prospective clinical studies and experiments *in vitro* and *in vivo* are needed to further clarify the physiological mechanism and provide clinical prospective.

In summary, our study suggested that circulating E2 played an important role in breast cancer etiology and was associated with increased risk even in low-estrogen nations with independent expression of ER status. In addition, higher circulating E2 were poor prognostic factor but leptin may be a protection signal in elderly postmenopausal Chinese patients with breast cancer.

## Data Availability Statement

The original contributions presented in the study are included in the article/supplementary material. Further inquiries can be directed to the corresponding author.

## Ethics Statement

The studies involving human participants were reviewed and approved by The Ethics Committee of the Cancer Hospital of the Chinese Academy of Medical Sciences. The patients/participants provided their written informed consent to participate in this study. Written informed consent was obtained from the individual(s) for the publication of any potentially identifiable images or data included in this article.

## Author Contributions

Y-QC designed and conceived the research and revised the manuscript. YL provided study materials and patients. H-BL drafted the manuscript. YZ analyzed and interpreted the data. Y-XW and DS optimized the experiment. All authors listed have contributed to the work and approved the final version.

## Funding

This study was funded by the National Natural Science Foundation of China (81972016) and the Chinese Academy of Medical Sciences Innovation Fund for Medical Sciences (Grant No. 2017-I2M-3-012 and No. 2017-I2M-1-013).

## Conflict of Interest

The authors declare that the research was conducted in the absence of any commercial or financial relationships that could be construed as a potential conflict of interest.

## Publisher’s Note

All claims expressed in this article are solely those of the authors and do not necessarily represent those of their affiliated organizations, or those of the publisher, the editors and the reviewers. Any product that may be evaluated in this article, or claim that may be made by its manufacturer, is not guaranteed or endorsed by the publisher.
